# Supplementary material for: Serum and Adipose Dipeptidyl Peptidase 4 in Cardiovascular Surgery Patients: Influence of Dipeptidyl Peptidase 4 Inhibitors
Source: J Clin Med. 2022 Jul 26;11(15):4333. doi: 10.3390/jcm11154333 (PMC9331841; doi:10.3390/jcm11154333)
Supplement: Supplementary file 1 [file jcm-11-04333-s001.zip › jcm-1790391-supplementary.pdf]

Supplementary Table S1. Characteristics of patients with and without atherosclerotic disease

	Atherosclerotic disease[a] (-)	Atherosclerotic disease[a] (+)	p-value[c]	Effect size[e]
Patients, Number	23	17		
Male, n (%)	16 (69.6)	9 (52.9)	0.283	
Age, years	64.2 ± 15.2	71.9 ± 10.0	0.062[d]	
BMI, kg/m[2]	24.8 ± 5.3	24.6 ± 2.6	0.914[d]	
Diagnosis				
NYHA	2.1 ± 1.2	2.1 ± 1.2	1.000[d]	
ECG				
Atrial fibrillation	10 (43.5)	1 (5.9)	0.008**	
Comorbidity				
Diabetes, n (%)	5 (21.7)	10 (58.8)	0.017*	
DPP-4 inhibitors, n (%)	4 (17.4)	6 (35.3)	0.196	
Hypertension, n (%)	16 (69.6)	13 (76.5)	0.629	
Dyslipidemia, n (%)	6 (26.1)	11 (64.7)	0.015*	
Smoking, n (%)	3 (13.0)	2 (11.8)	0.904	
Hemodialysis, n (%)	2 (8.7)	2 (11.8)	0.749	
Fasting blood glucose, mg/dL	110.0 ± 26.7	128.2 ± 39.3	0.173[d]	
Creatinine, mg/dL	1.27 ±1.27	1.22 ± 1.31	0.448[d]	
eGFR, mL/min/1.73 m[2]	61.3 ± 23.6	63.6 ± 27.1	0.665[d]	
Total cholesterol[b], mg/dL	178.3 ± 37.0	172.3 ± 43.6	0.944[d]	
High-Density Lipoprotein cholesterol, mg/dL	52.7 ± 12.8	50.5 ± 21.2	0.277[d]	
Low-Density Lipoprotein cholesterol, mg/dL	98.3 ± 27.5	97.9 ± 31.3	0.914[d]	
Triglycerides, mg/dL	112.0 ± 71.9	117.9 ± 64.1	0.432[d]	
Triglycerides/ High-Density Lipoprotein cholesterol	2.2 ± 1.5	2.7 ± 1.7	0.277[d]	
C-reactive protein, mg/dL	1.17 ± 2.07	0.65 ± 1.21	0.371[d]	
BNP, pg/mL	313.8 ± 411.6	351.7 ± 570.9	0.978[d]	
Hemoglobin A1c, %	6.01 ± 0.9	6.6 ± 0.9	0.024*[d]	
TTE				
AoD, mm	35.2 ± 9.0	28.6± 4.7	0.004**[d]	
LAD, mm	45.5 ± 9.5	42.5 ± 9.1	0.121[d]	
EF, %	55.8 ± 10.0	58.1± 9.5	0.665[d]	
Number of coronary artery lesions, vessel disease	0	1.8 ± 1.3	<0.001***[d]	
Serum levels				
Adiponectin, μg/ml	7.7 ± 6.6	11.5 ± 23.2	0.255[d]	γ=0.15

Leptin[b], pg/ml	5440.2 ± 5557.5	6081.1 ± 6691.3	0.978[d]	γ=0.06
DPP-4[b], ng/ml	166.1 ± 50.3	180.0 ± 65.7	0.851[d]	γ=0.13
DPP-4 activity, RFU/sec	3.8 ± 2.0	3.8 ± 1.2	0.808[d]	γ=0.02
Subcutaneous adipose				
DPP-4	0.87 ± 0.58	1.52 ± 0.71	0.004**[d]	γ=0.49
Adiponectin	1.55 ± 1.23	1.25± 0.87	0.289	γ=0.13
Epicardial adipose				
DPP-4	0.90 ± 0.62	0.80± 0.66	0.570[d]	γ=0.09
Adiponectin	0.56 ± 0.49	0.68 ± 0.57	0.273[d]	γ=0.12

The values are shown as mean ± SD. * <0.05, ** <0.01, and *** <0.001. [a]Atherosclerotic disease includes ischemic heart disease and aortic valve stenosis. [b]Missing values in total cholesterol (n=1), leptin (n=10), and DPP-4 (n=4) were excluded. [c]Using Chi-square test or Fisher's exact test. [d]Using Mann–Whitney U test. [e]Effect size was calculated by γ (≥0.1: small, ≥0.3: medium, ≥0.5: large) for Mann–Whitney. DPP-4, dipeptidyl peptidase 4; SD, standard deviation; BMI, body mass index; NYHA, New York Heart Association; ECG, electrocardiogram; BNP, brain natriuretic peptide; eGFR, estimated glomerular filtration; TTE, transesophageal echocardiography; AoD, aortic root diameter; LAD, left atrial dimension; EF, ejection fraction; RFU/s, relative fluorescent units per seconds.
